# Anterior Capsule Contrast and Capsulorhexis Geometry Across Visualization Systems in Cataract Surgery: An Exploratory Image-Based Comparison

**DOI:** 10.3390/jcm15197430

**Published:** 2026-09-24

**Authors:** Hisaharu Suzuki

**Affiliations:** Zengyo Suzuki Eye Clinic, Fujisawa 251-0871, Kanagawa, Japan; s5054@nms.ac.jp

**Keywords:** cataract surgery, continuous curvilinear capsulorhexis, digital visualization, heads-up display, NGENUITY, retrospective study

## Abstract

**Introduction**: Objective, image-based comparisons of anterior capsule visualization across conventional microscopy and digital heads-up visualization systems remain limited. This exploratory retrospective study quantitatively compared capsule contrast and capsulorhexis geometry among conventional microscopy and two successive generations of a digital heads-up system, both of which apply yellow digital filtering. **Methods**: This retrospective, non-randomized observational study included 67 eyes of 60 patients undergoing phacoemulsification using a conventional microscope (21 eyes) or NGENUITY with yellow filtering [version 1.4 (N1.4; 25 eyes) or 1.5 (N1.5; 21 eyes)], with visualization modality determined by clinical availability at the time of surgery. Color contrast ratio (CCR), ovality index, and concentricity were quantified from masked video analysis using linear mixed-effects models (patient as random intercept) with Holm–Bonferroni-adjusted comparisons. **Results**: Mean CCR was significantly higher in both NGENUITY groups than the Microscope group (N1.4: 1.55 ± 0.55; N1.5: 1.70 ± 0.29; Microscope: 1.14 ± 0.09; both *p* ≤ 0.008), with no difference between versions. The ovality index was significantly lower in the N1.5 group than the Microscope group (2.90 ± 1.78% vs. 5.08 ± 3.38%; *p* = 0.022), though this difference did not reach significance in the per-patient sensitivity analysis (*p* = 0.071); no significant difference was observed between the Microscope and N1.4 groups. Concentricity did not differ significantly among groups. **Conclusions**: Anterior capsule contrast, as captured in recorded surgical video, was objectively higher with NGENUITY than with conventional microscopy, whereas a modest improvement in capsulorhexis circularity was observed only with version 1.5. These exploratory, hypothesis-generating findings are intended to motivate, rather than substitute for, a prospective, randomized, within-system study; they provide a standardized measurement framework showing that contrast parameters differ across visualization modalities, while geometric advantages across system generations warrant confirmation in such future studies.

## 1. Introduction

Continuous curvilinear capsulorhexis (CCC) is one of the most critical steps in modern cataract surgery. Its size, shape, and centration directly influence intraocular lens stability, effective lens position, and long-term refractive outcomes [1,2,3,4]. Precise identification of the anterior capsule edge is therefore essential for achieving a well-centered, circular capsulotomy.

In recent years, digitally assisted three-dimensional (3D) heads-up visualization systems have been increasingly adopted in cataract surgery. Compared with conventional optical microscopy, these systems enable real-time adjustment of brightness, contrast, color balance, gamma correction, and spectral filtering [5,6,7]. Previous clinical studies have demonstrated surgical safety and efficiency comparable with those of conventional microscopy, along with the advantage of lower intraoperative illumination levels [6,7]. These developments have shifted attention from simply replacing the conventional microscope to optimizing digital visualization itself. The NGENUITY^®^ 3D Visualization System (Alcon Vision LLC, Fort Worth, TX, USA) exemplifies this approach, enabling real-time adjustment of digital color parameters—including hue, saturation, gamma, and channel weighting—to modify the intraoperative appearance of ocular tissues [8,9,10,11,12].

Among currently available image-processing options, yellow digital filtering has attracted increasing interest because it selectively attenuates short-wavelength blue light while preserving natural color information. This spectral modification may reduce intraocular light scatter and chromatic aberration while improving luminance contrast between transparent ocular tissues and the red reflex [13,14,15]. Unlike monochrome filters, which may impair stereopsis and compromise visibility of hemorrhagic tissues, yellow filtering maintains stereoscopic color perception and may facilitate delicate intraocular maneuvers requiring accurate depth perception [12,16].

Although previous studies have described the feasibility and potential advantages of digital filters during cataract surgery [8] and confirmed the overall safety and efficacy of three-dimensional visualization systems [6,7], objective quantitative evidence demonstrating how yellow digital filtering influences anterior capsule visualization remains limited. No previous study has simultaneously quantified anterior capsule contrast and capsulorhexis geometry using standardized image-based analysis of surgical video. Intraoperative image analysis provides a standardized means of quantifying recorded image characteristics; parameters such as the color contrast ratio (CCR) between the capsulotomy edge and the adjacent anterior capsule and geometric indices of capsulotomy morphology can be extracted from recorded surgical video and analyzed in a standardized, masked fashion [17,18].

Therefore, the purpose of the present study was to perform an exploratory, image-based comparison of conventional microscopy and two successive versions of the NGENUITY heads-up system, both applying yellow digital filtering, by quantitatively analyzing CCR, capsulorhexis ovality, and capsulorhexis concentricity under routine surgical conditions. Because the visualization systems compared differ in several respects beyond the filter itself—including the imaging pipeline, display, and, between NGENUITY versions, software generation—this study was not designed to isolate the independent contribution of yellow filtering per se; rather, we sought to generate a descriptive, hypothesis-generating account of whether objective anterior capsule contrast and capsulorhexis geometry differ across these visualization modalities as used in clinical practice, to inform the design of future prospective, within-system studies that can isolate the filter’s independent effect.

## 2. Methods

### 2.1. Three-Dimensional Heads-Up Visualization System and the Conventional Surgical Microscope

The 3D heads-up visualization system used was NGENUITY, which consists of an HDR camera and a 55-inch OLED 4K display, connected to an HS Hi-R NEO 900 surgical microscope (Haag-Streit AG, Köniz, Switzerland), which was also used independently as the conventional microscope. This system enables real-time adjustment of RGB channels, hue, saturation, and gamma to modify intraoperative color characteristics. Compared with version 1.4, version 1.5 introduced several additional image-processing functions, including NGENUITY Factor for image sharpening, Blue Boost for modifying the visibility of yellowish tissues, and Performance Green for modifying the color balance, contrast, and luminance of vitreous and trabecular structures.

The conventional microscope, HS Hi-R NEO 900 (Haag-Streit AG, Koeniz, Switzerland), is a high-resolution optical system with a wide field of view and deep depth of focus, equipped with an LED light source (color temperature approximately 4500 K) and coaxial illumination with red reflex enhancement for stable visualization during CCC. Because of the optical design of this platform, NGENUITY cannot be connected while the microscope’s conventional ocular/tube unit is in place; using NGENUITY requires removing the microscope’s optical tube assembly and mounting the NGENUITY camera in its place. Consequently, it is mechanically impossible to record the Microscope and NGENUITY groups through a single, shared imaging and recording system—an inherent structural constraint of comparing these two visualization modalities in the same clinical setting, rather than a methodological choice, and one that necessitated the two distinct recording pathways described in Section 2.4.

### 2.2. Study Design and Ethics

This single-center retrospective observational study was approved by the institutional review board (approval number: 21000029) and adhered to the tenets of the Declaration of Helsinki. Given the retrospective design and use of routinely collected surgical video data, the requirement for individual informed consent was waived by the Ethics Committee; patient anonymity was protected through an opt-out procedure.

### 2.3. Participants and Case Selection

Adult patients (aged 50–90 years) who underwent routine phacoemulsification with monofocal IOL implantation between December 2020 and March 2024 were retrospectively screened from electronic medical records. A total of 97 eyes were initially assessed for eligibility (31 eyes in the Microscope group, 35 in the N1.4 group, and 31 in the N1.5 group). Cases with insufficient image quality—objectively defined as inability to extract accurate RGB values at the predefined region of interest (330–360°) due to technical factors including out-of-focus frames, excessive specular reflection, or instrument obscuration—were excluded before protocol eligibility assessment by an investigator not involved in image analysis. Across all modalities, 30 eyes were excluded before the final analysis for this and other protocol-defined reasons (10 eyes in each group; exclusion rates 32.3%, 28.6%, and 32.3% in the Microscope, N1.4, and N1.5 groups, respectively; chi-square test, *p* = 0.931, indicating no significant difference in exclusion rates across modalities). A total of 67 eyes (21 Microscope, 25 N1.4, 21 N1.5) were included in the final statistical analysis. Three visualization modalities were compared: conventional microscopy (Microscope group), NGENUITY version 1.4 (N1.4 group), and NGENUITY version 1.5 (N1.5 group), assigned according to the system in clinical use at the time of surgery (not randomized).

Inclusion criteria were: Emery–Little cataract grade 1, 2, or 3; and CCC diameter ≤5.3 mm (to minimize geometric variability attributable to capsulotomy size). Exclusion criteria were: posterior subcapsular cataract, corneal opacity affecting the red reflex, inadequate pupil dilation precluding full visualization of the IOL optic edge, axial length >26 mm, prior ocular surgery, pseudoexfoliation syndrome, zonular weakness, and any intraoperative complication. These criteria were intentionally applied to reduce confounding variability in image-based parameters attributable to factors other than visualization modality, so that the resulting cohort represents routine cataract cases in which both conventional microscopy and digital visualization are used clinically. The implanted IOL model varied across cases, but the IOL optic diameter was uniform (6.0 mm) in every case, and the same ophthalmic viscosurgical device (DisCoVisc, Alcon Vision LLC, Fort Worth, TX, USA) was used throughout the study period. Pupil diameter was not fixed or identical across cases; all included eyes, however, satisfied the inclusion criterion that the pupil margin extended beyond the IOL optic edge, and eyes with pupil dilation inadequate for full visualization of the IOL optic edge were excluded, as noted above.

Archived surgical videos of eligible patients were retrieved for image analysis. Cases with insufficient image quality, defined as the inability to extract accurate RGB values at the region of interest (330–360°) due to out-of-focus frames, excessive specular reflection, or instrument obscuration, were excluded.

### 2.4. Surgical Technique and Visualization Settings

All procedures were performed by a single experienced surgeon using a standardized phacoemulsification technique. A 2.4 mm slit knife (MANI Inc., Tochigi, Japan) was used to create a transconjunctival single-plane sclerocorneal incision. CCC with a target diameter of 5.0 mm was created using Inamura capsulorhexis forceps (fine titanium; Inami & Co., Ltd., Tokyo, Japan) without viscoelastic staining agents; trypan blue capsular staining was not used in any group, so all capsulorhexes relied on the unstained red reflex for visualization. Lens removal was performed with a CENTURION^®^ VISION SYSTEM (Alcon Vision LLC, Fort Worth, TX, USA) equipped with a 0.9 mm 45-degree ABS Balanced Tip and UltraSleeve. The IOL model implanted varied across cases, but all implanted IOLs had a uniform 6.0 mm optic diameter, and the same viscoelastic device (DisCoVisc, Alcon Vision LLC, Fort Worth, TX, USA) was used throughout.

For conventional microscopy, the HS Hi-R NEO 900 was used with an LED light source at 50% illumination intensity. Video for the Microscope group was captured via a beam splitter (Haag-Streit, model 657240; light-distribution ratio 80:20) feeding a dedicated recording camera (Panasonic GP-UH332; 1920 × 1080 resolution, 50 p frame rate), connected to a medical-grade recorder (Ikegami MDR-600HD; H.264/MPEG-4 AVC encoding, .mov container, approximately 18 Mbps at the SQ setting used, 8-bit depth). White balance was set automatically prior to each case and held fixed thereafter; exposure was automatic; gain was automatic or manually adjustable via the illumination-level control; aperture was manually adjusted via the camera adapter and the microscope’s double-iris diaphragm. No HDR processing was applied, and no post-recording re-compression, transcoding, LUT-based gamma remapping, color correction, or file rewrapping was performed. For digital visualization, NGENUITY was connected to the same microscope body (in place of the conventional ocular/tube unit, as described in Section 2.1), and video for the N1.4 and N1.5 groups was recorded directly from the NGENUITY system’s native digital output, via a pathway necessarily separate from the Microscope group’s beam-splitter/camera/recorder chain. The display was positioned approximately 1.2 m directly in front of the operator, who wore circular polarizing glasses for stereoscopic visualization. The iris diaphragm of the digital camera was set to approximately 30% aperture. Yellow digital filtering was applied during CCC creation in both NGENUITY groups; all other NGENUITY parameters were constant between N1.4 and N1.5 (Table 1). The only parametric difference between versions was the NGENUITY Factor setting (set to 3 in N1.5), which provides additional image contrast enhancement through digital processing of luminance gradients.

### 2.5. Image-Based Outcome Measures

#### 2.5.1. Color Contrast Ratio

Representative still frames capturing completed CCC were extracted from surgical video at a standardized export resolution. Image extraction and analysis were performed by a third-party examiner masked to group assignment. Two regions of interest were defined for each image—the CCC edge and the adjacent anterior capsule background—consistently at positions between 330° and 360°, and analyzed using DIPP-Motion V (version 1.2.5; DITECT, Tokyo, Japan) (Figure 1). This sector was selected because it consistently provided the most stable visualization of the CCC edge and adjacent anterior capsule across cases, minimizing interference from surgical instruments or the corneal incision.

Each region of interest (ROI) was defined not as a single pixel, but as a 2 × 2 pixel area, and the average RGB values within this region were extracted. Because the CCC edge is an extremely fine structure, this 2 × 2 pixel size was selected to faithfully capture the delicate edge without excessively incorporating surrounding tissue, while suppressing single-pixel noise. For each of the three repeated measurements, the examiner independently repositioned the 2 × 2 pixel ROI within the 330–360° sector rather than reusing fixed coordinates.

The color luminance (CL) and CCR were calculated according to the report by Kadonosono et al. [17]. First, the extracted R, G, and B values (8-bit, range 0–255) were normalized to the range [0, 1] by dividing by 255. If the normalized R was ≤0.03928, Rs was estimated using Rs = R/12.92, whereas if R was >0.03928, it was estimated using Rs = [(R + 0.055)/1.055]^2.4^; Gs and Bs were estimated identically from the normalized G and B values. The CL was estimated as L = 0.2126Rs + 0.7152Gs + 0.0722Bs. The CCR was calculated as CCR = (Lmax + 0.05)/(Lmin + 0.05), where Lmax is the luminance of the brighter background and Lmin is the luminance of the darker background. CCR was measured three times per image and the mean value was used for analysis; intraobserver repeatability (intraclass correlation coefficient and coefficient of variation) was assessed from these triplicate measurements and is reported in Section 3.2.

#### 2.5.2. Capsulorhexis Geometry

All geometric evaluations were conducted by an independent examiner using surgical videos, following previously reported methodologies [18]. Ovality index was calculated as: (long axis − short axis)/[(long axis + short axis)/2] × 100 (%); a lower value indicates a more circular capsulotomy. CCC concentricity was assessed on frames in which both the IOL optic edge and CCC margin were identifiable (Figure 2), calculated as (Omax − Omin)/2, where Omax and Omin represent the maximum and minimum overlap of the capsulotomy over the IOL optic edge; a lower value indicates better centration. Pixel-to-micrometre conversion was calibrated using the IOL optic diameter (6.0 mm, uniform across all cases regardless of IOL model) as the internal reference scale. No post hoc digital correction for camera angle, globe rotation, or perspective distortion was applied; however, intraoperative axial alignment was inherently self-corrected, as the surgeon continuously verified a near-perpendicular viewing axis by monitoring the symmetry and completeness of the red reflex (retroillumination) from the fundus—standard intraoperative practice during phacoemulsification. Correspondingly, the representative frame used for each geometric measurement was the frame demonstrating the clearest, most symmetric and complete red reflex, corresponding to the most axially aligned, best-focused view available. Geometric measurements were performed by the same single, masked examiner as the CCR measurements; a second examiner was not involved, and formal interobserver agreement for the geometric measures was not assessed (Section 4).

### 2.6. Baseline Characteristics

To assess between-group comparability, the following preoperative characteristics were recorded for each case: age, sex, Emery–Little cataract grade, and preoperative best-corrected visual acuity (BCVA).

### 2.7. Sample Size and Statistical Methods

Sample size estimation was based on an independent pilot dataset (n = 5 per group): mean CCR of 1.16 ± 0.08 (Microscope), 1.55 ± 0.21 (N1.4), and 1.75 ± 0.34 (N1.5). Power analysis using a one-way ANOVA framework (two-sided α = 0.05) indicated that 16 eyes per group would yield power exceeding 90% for the anticipated CCR difference; the final sample of 21–25 eyes per group exceeded this minimum. We acknowledge that this a priori power calculation used a simpler one-way ANOVA framework than the linear mixed-effects model (LMEM) ultimately specified as the primary analysis to account for bilateral-eye correlation; the LMEM nonetheless provided adequate power to detect the observed primary effects at conventional significance thresholds.

Continuous variables are reported as mean ± SD. Normality was assessed using the Shapiro–Wilk test. Because nine patients contributed data from both eyes (five in the Microscope group, one in the N1.4 group, one in the N1.5 group, and two contributing eyes across groups), strict statistical independence between observations cannot be assumed. In this study, CCR was defined as the primary outcome, whereas capsulorhexis geometry parameters (ovality and concentricity) served as secondary outcomes. Between-group comparisons of all these outcomes were therefore performed using linear mixed-effects models (LMEMs) with patient as a random intercept and visualization group as a fixed effect; model fit was assessed by likelihood ratio test against a null model. Pairwise post hoc comparisons used estimated marginal means with Holm–Bonferroni correction; effect sizes were quantified using Cohen’s d. As a sensitivity analysis, one eye was randomly selected per patient (seed = 123) and outcomes were re-evaluated using one-way ANOVA. All analyses were performed using R version 4.3.3 (R Foundation for Statistical Computing, Vienna, Austria) with the lme4 and emmeans packages. All tests were two-sided, with *p* < 0.05 considered statistically significant. Cohen’s d was calculated as the raw, unadjusted eye-level mean difference between groups divided by the pooled standard deviation of the two groups being compared [d = (mean2 − mean1)/√((SD1^2^ + SD2^2^)/2)], and is reported separately from the LMEM-derived mean-difference estimates, *p*-values, and z-statistics, which account for patient-level clustering. As additional sensitivity analyses, we refit the CCR model with Emery–Little grade included as a fixed-effect covariate; the between-group differences remained significant and were, if anything, slightly larger after adjustment (Microscope vs. N1.4: *p* = 0.0013; Microscope vs. N1.5: *p* < 0.0001), indicating that nuclear grade does not account for the observed CCR differences. Because Brown–Forsythe testing indicated significant heterogeneity of CCR variance across groups (*p* = 0.020), we further refit the model allowing group-specific residual variances (heteroscedastic mixed model; R package nlme; weights = varIdent (form = ~1|group)); this model fit the data substantially better (likelihood ratio test *p* < 0.0001) and the between-group differences remained significant (both *p* < 0.001), indicating that the primary conclusions are robust to this variance heterogeneity. 

Claude (Anthropic; https://claude.ai/; accessed on 23 September 2026) and ChatGPT (OpenAI; https://chatgpt.com/; accessed on 23 September 2026) were used to assist with English translation and grammatical editing. Claude was also used to support the implementation of the linear mixed-effects statistical models. The author reviewed and verified all AI-assisted work.

## 3. Results

### 3.1. Study Population and Baseline Characteristics

A total of 67 eyes from 60 patients were included: 21 eyes in the Microscope group, 25 in the N1.4 group, and 21 in the N1.5 group. Baseline characteristics are summarized in Table 2; no significant differences were observed among groups in age, sex, Emery–Little grade, or preoperative BCVA (all *p* > 0.05). No intraoperative CCC-related complications occurred, and all capsulorhexes remained completely within the optic margin of the implanted IOL.

### 3.2. Color Contrast Ratio

Mean CCR was 1.14 ± 0.09 in the Microscope group, 1.55 ± 0.55 in the N1.4 group, and 1.70 ± 0.29 in the N1.5 group (overall *p* < 0.001). In the primary LMEM analysis, the N1.4 and N1.5 groups showed significantly higher CCR than the Microscope group (estimated mean difference: Microscope vs. N1.4, 0.34, Cohen’s d = 1.03, z = 2.86, *p* = 0.008; Microscope vs. N1.5, 0.42, Cohen’s d = 2.58, z = 4.12, *p* < 0.001). CCR did not differ significantly between N1.4 and N1.5 (*p* = 0.51) (Figure 3). The sensitivity analysis using one eye per patient yielded consistent results (Microscope vs. N1.4: *p* = 0.006; Microscope vs. N1.5: *p* < 0.001).

Intraobserver repeatability of the triplicate CCR measurements was assessed using two-way random-effects intraclass correlation coefficients: ICC(3, 1), reflecting the reliability of a single measurement, and ICC(3, k), reflecting the reliability of the mean of three measurements used as the final reported CCR value. ICC(3, 1) was 0.629 (95% CI 0.396–0.811) in the Microscope group, 0.942 (95% CI 0.893–0.972) in the N1.4 group, and 0.779 (95% CI 0.607–0.894) in the N1.5 group; the corresponding ICC(3, k) values were 0.836 (95% CI 0.663–0.928), 0.980 (95% CI 0.961–0.991), and 0.913 (95% CI 0.823–0.962), respectively, indicating good-to-excellent reliability of the averaged CCR value used for analysis. Coefficients of variation (CoV) were 4.42%, 7.41%, and 7.31%, respectively.

### 3.3. Capsulorhexis Geometry

Mean ovality indices were 5.08 ± 3.38% (Microscope), 4.19 ± 2.72% (N1.4), and 2.90 ± 1.78% (N1.5) (Figure 4). LMEM analysis showed a significantly lower ovality index in the N1.5 group than the Microscope group (estimated mean difference −2.18%, Cohen’s d = −0.81, z = −2.68, *p* = 0.022); no significant differences were observed between the Microscope and N1.4 groups (*p* = 0.25) or between the two NGENUITY versions (*p* = 0.20). In the sensitivity analysis, the same directional trend was observed, but the Microscope vs. N1.5 difference did not reach significance (*p* = 0.071), consistent with reduced power.

Mean CCC concentricity was comparable among groups (Microscope: 55.27 ± 43.63 μm; N1.4: 52.61 ± 33.99 μm; N1.5: 52.70 ± 40.04 μm), with no significant between-group differences (all *p* = 1.00).

## 4. Discussion

This is a retrospective, non-randomized, single-center, exploratory comparison, and the findings below should be interpreted accordingly: our purpose is to generate a standardized, quantitative signal motivating future prospective, randomized, within-system research, not to establish causal or generalizable claims about the NGENUITY system.

The present study provides an objective, image-based quantitative comparison of conventional microscopy and a digital heads-up visualization system incorporating yellow digital filtering during cataract surgery. To our knowledge, this is the first study to simultaneously quantify anterior capsule contrast and capsulorhexis geometry using standardized, masked image analysis of surgical video, providing a standardized approach to post hoc analysis of recorded surgical video.

Using this framework, anterior capsule contrast in the recorded video images was objectively higher in both NGENUITY groups than with conventional microscopy, with large effect sizes, whereas a lower ovality index—indicating greater circularity—was observed only in the N1.5 group; CCC concentricity did not differ significantly among groups. Because conventional microscopy and the NGENUITY system differ in multiple respects beyond the yellow filter—including the image-acquisition pipeline, display, and, between software versions, additional processing features—these findings describe an association between visualization modality and objective image parameters rather than a causal, isolated effect of yellow filtering; this distinction is addressed further below and in the Limitations.

One plausible contributor to the higher CCR observed in the NGENUITY groups is yellow digital filtering itself, which selectively attenuates short-wavelength blue light, a major contributor to intraocular light scatter and chromatic aberration [13]; by reducing these optical disturbances while preserving luminance gradients, yellow filtering could plausibly enhance apparent contrast between the capsulotomy edge and anterior capsule background. This candidate mechanism is consistent with prior work demonstrating that yellow filters improve contrast sensitivity under glare conditions in healthy eyes [14] and in high myopes [15]. However, because the present comparison also involved differences in camera sensor, digital signal processing, and display between the microscope and NGENUITY groups, other elements of the digital imaging pipeline may have contributed independently to the observed CCR difference, and the relative contribution of the yellow filter specifically cannot be isolated from the present data. If yellow filtering does contribute to the observed contrast enhancement, it would do so while preserving stereoscopic color perception, unlike monochrome filters, potentially maintaining the depth perception required for precise anterior segment maneuvers [12,16]; this remains to be confirmed in studies that isolate the filter within a single imaging platform.

This CCR methodology was adapted from objective image-analysis techniques originally developed for vitreoretinal surgery [17], and its extension here to quantitative, masked assessment of anterior capsule contrast during cataract surgery is, to our knowledge, novel and readily transferable to future comparisons of any visualization system. The CCR values observed in both NGENUITY groups are consistent with those reported by Sandali et al., who demonstrated increased contrast ratios using the monochrome mode of the NGENUITY system [12]; unlike monochrome filtering, however, yellow filtering preserves stereoscopic color cues.

Although CCR tended to be higher in the N1.5 group than the N1.4 group, this difference was not statistically significant after correction (*p* = 0.52). Because software version and associated image-processing functions changed simultaneously between study periods, the independent contribution of the NGENUITY Factor could not be isolated; prospective studies using identical hardware with isolated software modifications would be needed to clarify this. CCR and ovality are conceptually distinct outcomes measuring different aspects of visualization quality; the NGENUITY Factor image-sharpening feature unique to N1.5 (Table 1) may plausibly aid edge-following behavior during capsulorhexis creation without proportionally affecting the color-contrast metric, but this candidate explanation is post hoc and unconfirmed, and the overall pattern should be regarded as hypothesis-generating.

The observed difference in ovality index between the N1.5 and Microscope groups (2.90 ± 1.78% vs. 5.08 ± 3.38%) is of potential clinical interest and numerically comparable with that reported for the Zepto Precision Pulse Capsulotomy device (3.0 ± 2.86%), an automated system designed to produce circular capsular openings [18]. Whether this reflects enhanced visualization or stochastic variation cannot be determined from the present data; the corresponding sensitivity analysis did not reach significance (*p* = 0.071), consistent with limited power. Nonetheless, the primary LMEM result (*p* = 0.022) suggests that digital image processing affects quantifiable aspects of capsulotomy geometry and merits evaluation in larger prospective studies. Beyond digital visualization filters and automated capsulotomy devices, a broader body of work has pursued objective, automated assessment of the capsulorhexis itself; for example, Lin et al. developed a neural-network model that detects the capsular tear and lens margin from images and computes standardized circularity and eccentricity indices, validated on an artificial-eye phantom [19]. Our image-based CCR and geometry framework is complementary to such approaches: rather than assisting or automating capsulotomy creation, it provides a standardized, masked, post hoc method for quantifying image-based differences associated with the visualization system and the surgeon’s manually created capsulotomy, and could in principle be paired with automated detection pipelines to reduce examiner-dependent variability in future studies.

The absence of a significant difference in CCC concentricity suggests that experienced surgeons can achieve excellent centration irrespective of visualization modality. All three groups achieved better centration than reported for the Zepto device (197 ± 122 μm) [18], suggesting that manual CCC by an experienced surgeon provides high centration precision regardless of the visualization system; direct comparison with automated systems should be interpreted cautiously given differences in design and methodology.

This study has several important limitations. Its retrospective, single-center, single-surgeon design and non-randomized allocation—group assignment followed clinical equipment availability—confound visualization modality with the time period of surgery, so secular changes cannot be excluded. More fundamentally, conventional microscopy and the NGENUITY system differ not only in the yellow filter but also in camera sensor, digital signal processing, display, and, between N1.4 and N1.5, software-level processing; because a within-system filter on/off comparison was not performed, the observed differences in CCR and geometry reflect visualization modality as a whole rather than the yellow filter in isolation. Furthermore, because video export resolution and compression were not independently equalized, and conventional microscopy relies on a beam-splitter camera rather than NGENUITY’s direct digital capture, the quantified CCR reflects recorded image contrast. Therefore, the lower CCR in the Microscope group may partly reflect these recording pathway differences and may not fully correspond to the surgeon’s real-time visual perception. Technically challenging cases (posterior subcapsular cataract, poor red reflex, small pupil) were excluded to minimize confounding; the findings therefore describe a baseline under favorable visualization conditions and do not address the more clinically relevant scenario of reduced media clarity, where digital enhancement might matter most. Finally, this study is limited to intraoperative image-based parameters; associations with surgical performance and postoperative outcomes (IOL centration, refraction, complications) were outside its scope, and CCR, ovality, and concentricity should be regarded as surrogate rather than clinical endpoints. As a structural constraint of this comparison, NGENUITY cannot be physically connected while the microscope’s conventional ocular/tube unit is in place (Section 2.1), so the two modalities necessarily used distinct recording pathways; this is an inherent limitation of comparing these visualization systems rather than a design choice. Geometric measurements were performed by a single examiner without a second examiner for interobserver comparison. Exclusion criteria based on CCC diameter (>5.3 mm) and intraoperative complications, while intended to reduce confounding variability unrelated to visualization modality, necessarily restrict our findings to eyes in which a technically successful, appropriately sized capsulorhexis was achieved, and results should not be extrapolated to more technically challenging cases.

Bilateral non-independence was addressed using LMEMs, but only nine patients contributed bilateral data, limiting the precision of the random-effect estimate; the per-patient sensitivity analysis was consistent for CCR, though the Microscope-vs-N1.5 ovality difference lost significance (*p* = 0.071), reflecting reduced power rather than a change in effect direction. Software updates between the N1.4 and N1.5 periods preclude isolating individual processing functions. This study was supported by an investigator-initiated grant from the NGENUITY manufacturer, which, together with its exploratory, non-randomized design, warrants cautious interpretation. Future work should adopt a prospective, within-system design randomizing yellow filtering on/off under identical hardware, software, and recording conditions—including eyes with reduced media clarity—to isolate the filter’s contribution and link objective parameters to clinical outcomes.

In this exploratory retrospective comparison, objective anterior capsule contrast in recorded intraoperative video was higher with the NGENUITY heads-up visualization system, which incorporates yellow digital filtering, than with conventional microscopy in routine cataract surgery; a modest improvement in capsulorhexis circularity was additionally observed in the N1.5 subgroup only. Because visualization modalities differed across multiple dimensions of the imaging pipeline rather than the yellow filter alone, and because the ovality finding did not reach significance in the per-patient sensitivity analysis, these findings should be regarded as hypothesis-generating rather than confirmatory of a specific filter effect or a system-wide geometric benefit. More broadly, by applying a standardized, masked framework for quantifying recorded intraoperative image characteristics, this study provides an exploratory basis for future prospective, within-system studies to determine whether these image-based observations translate into better surgical performance and long-term clinical outcomes.

## Figures and Tables

**Figure 1:**
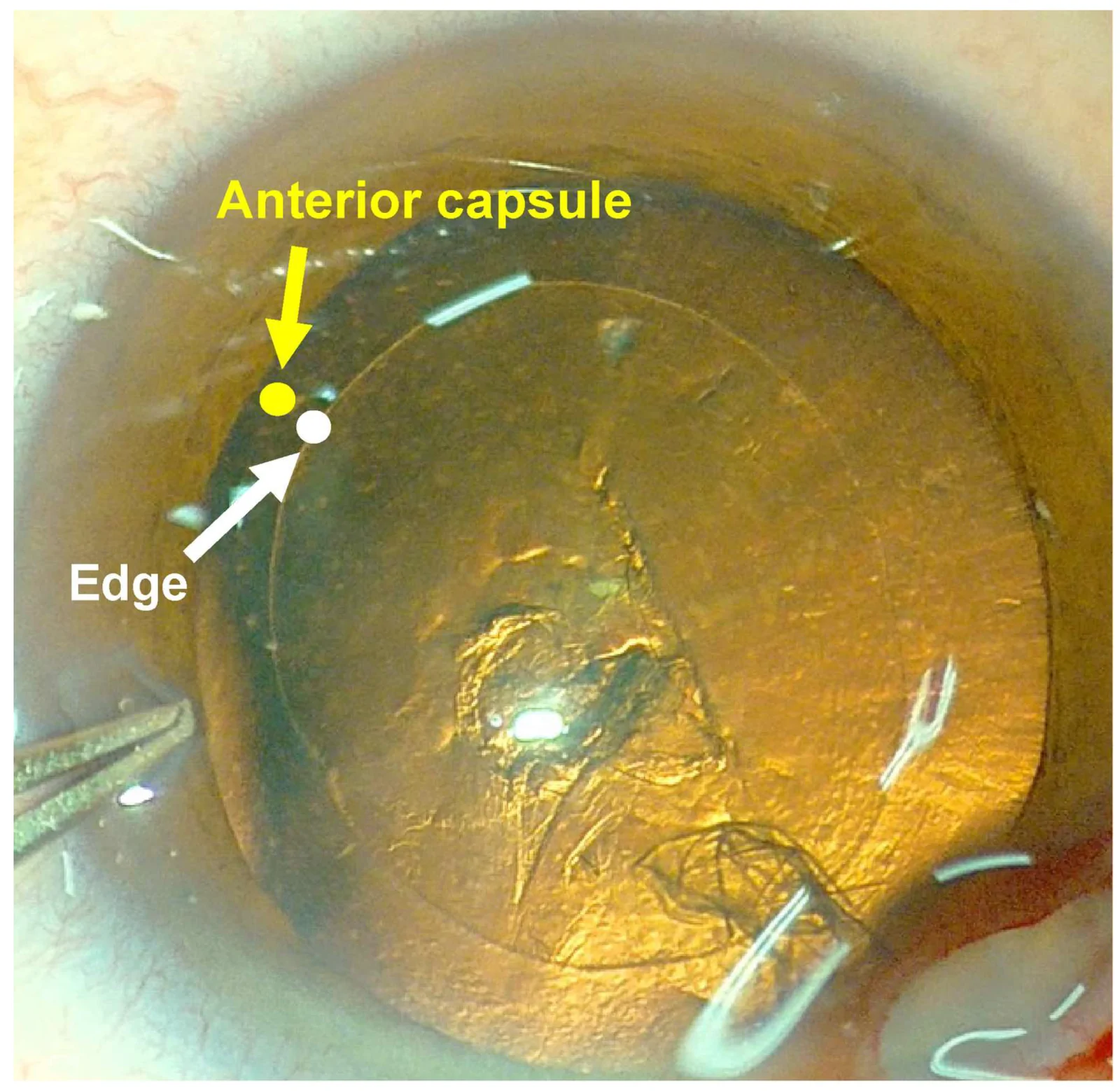
Representative intraoperative video frame illustrating the regions of interest for CCR measurement. The white circle marks the CCC edge (measurement region between 330° and 360°); the yellow circle indicates the adjacent anterior capsule area. RGB values from these two regions are used to calculate luminance and CCR. CCR = color contrast ratio; CCC = continuous curvilinear capsulorhexis.

**Figure 2:**
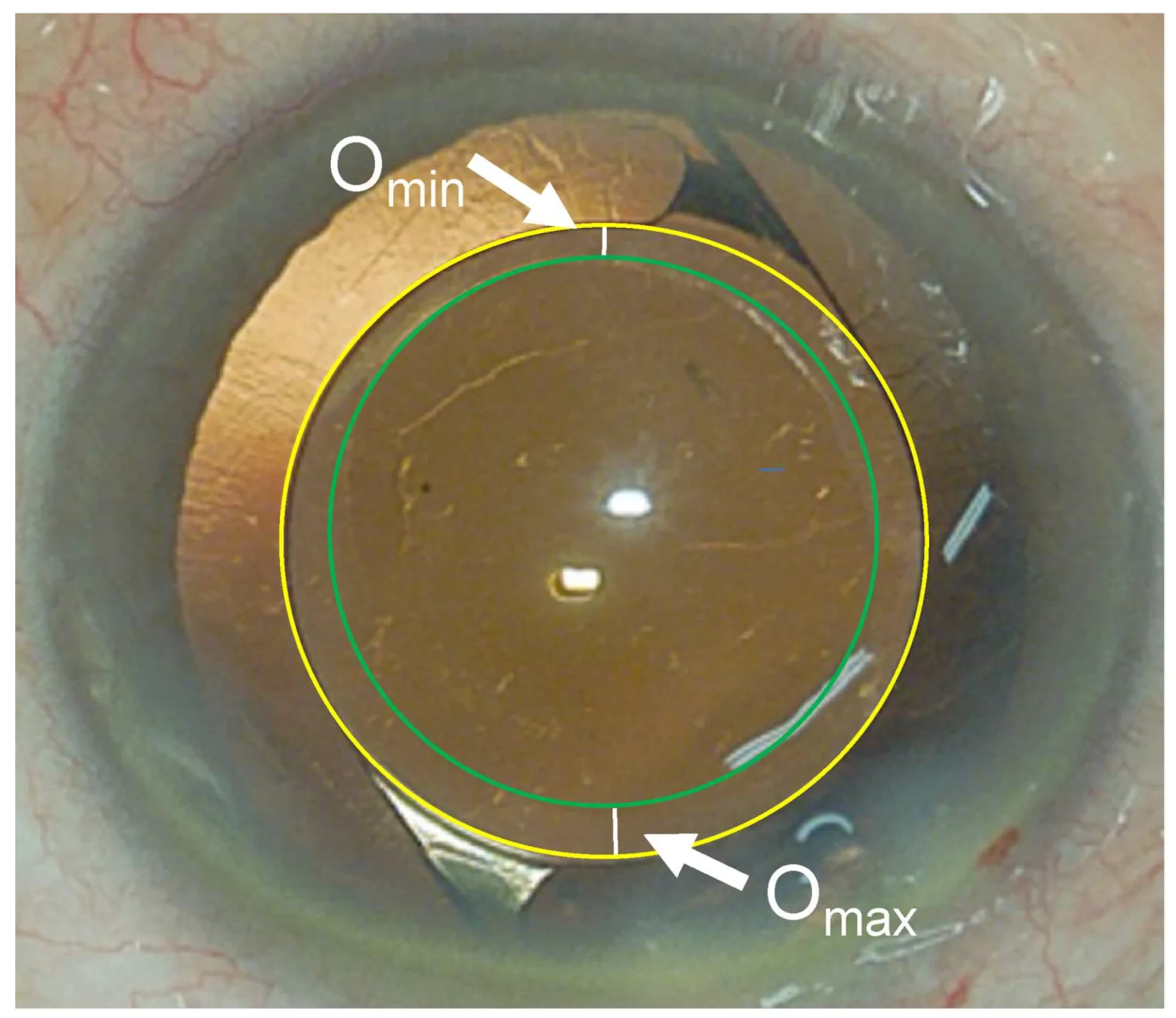
Schematic diagram illustrating the measurement of CCC concentricity. Omax and Omin represent the maximum and minimum overlap of the capsulotomy edge over the IOL optic margin, respectively. Concentricity is calculated as (Omax − Omin)/2. IOL = intraocular lens; CCC = continuous curvilinear capsulorhexis. The yellow outline marks the IOL optic margin, and the green outline marks the CCC edge.

**Figure 3:**
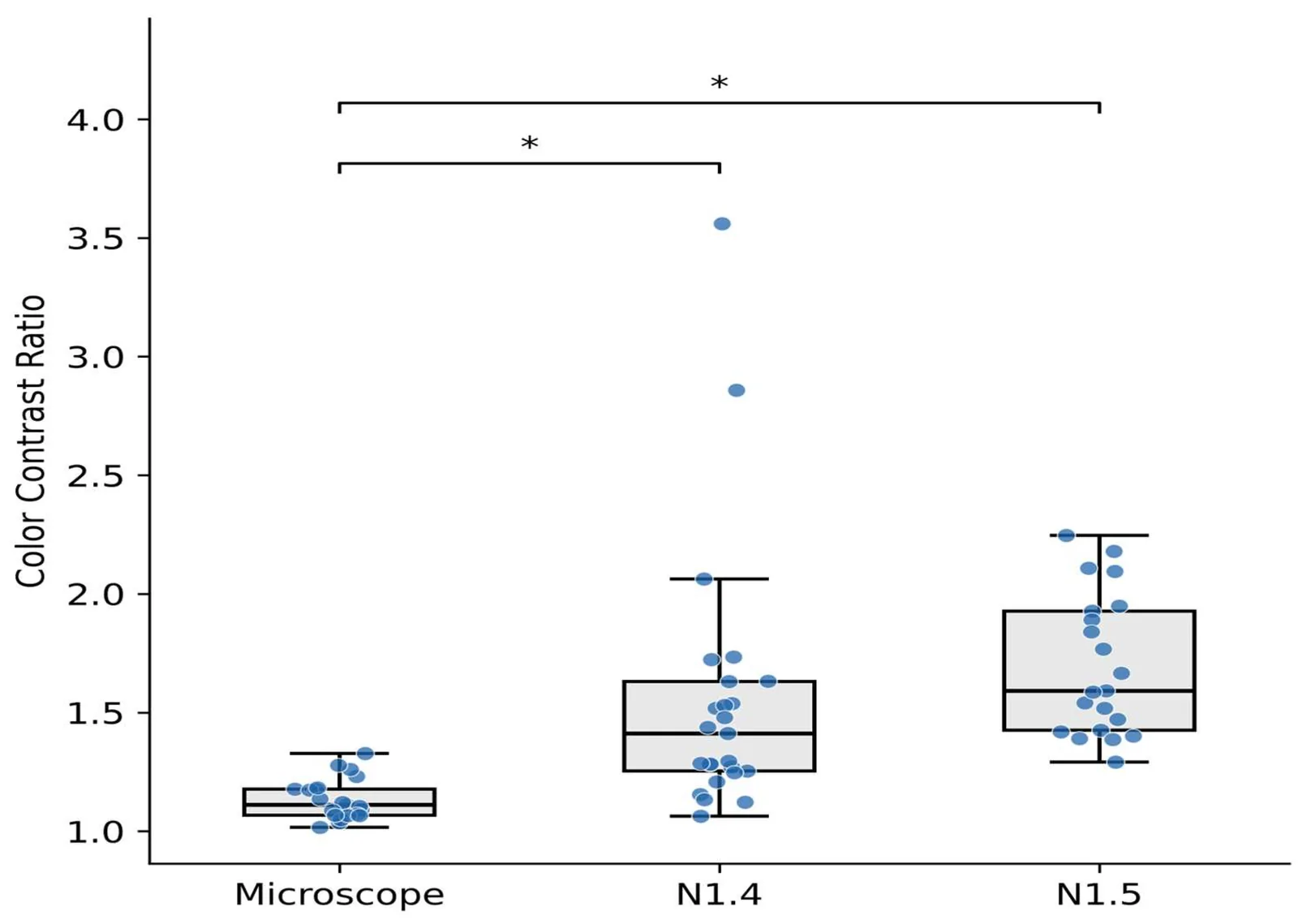
Comparison of CCR among the Microscope, N1.4, and N1.5 groups, shown as box-and-dot plots with individual eye-level observations (median, interquartile range, and range). * *p* ≤ 0.008 vs. Microscope group (linear mixed-effects model with Holm–Bonferroni correction). CCR = color contrast ratio; N1.4 = NGENUITY version 1.4; N1.5 = NGENUITY version 1.5.

**Figure 4:**
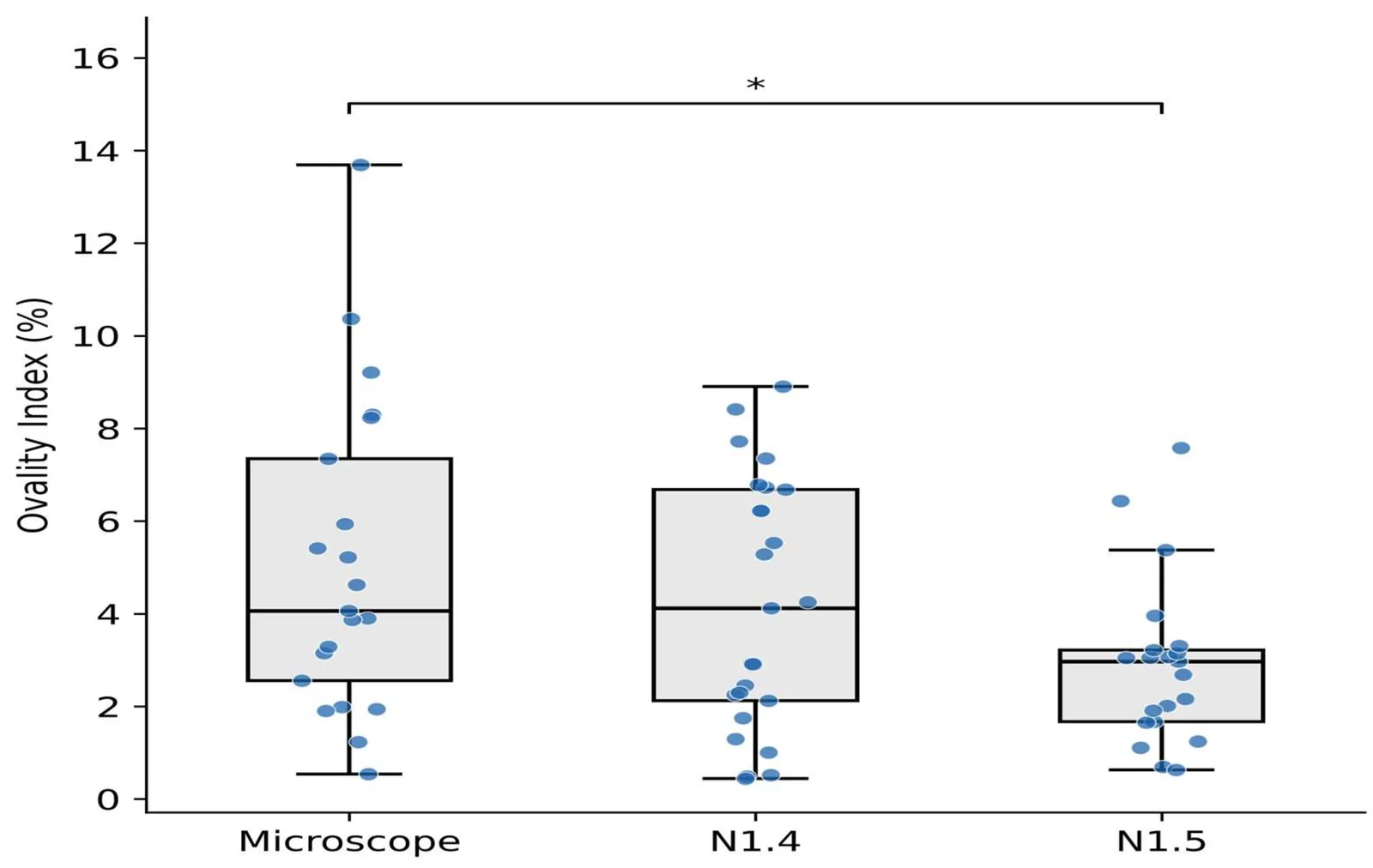
Ovality index (%) among the Microscope, N1.4, and N1.5 groups, shown as box-and-dot plots with individual eye-level observations; lower values indicate greater circularity. * *p* = 0.022 for Microscope vs. N1.5 (linear mixed-effects model with Holm–Bonferroni correction). CCC concentricity did not differ significantly among groups (Microscope: 55.27 ± 43.63 µm; N1.4: 52.61 ± 33.99 µm; N1.5: 52.70 ± 40.04 µm; all *p* = 1.00). N1.4 = NGENUITY version 1.4; N1.5 = NGENUITY version 1.5; CCC = continuous curvilinear capsulorhexis.

**Table 1:** NGENUITY System Settings for N1.4 and N1.5 Groups.

Parameter	N1.4	N1.5
Iris slider (%)	70	70
Gain	6	6
Red/Green/Blue	86/100/94	86/100/94
Brightness	47.4	47.4
Contrast	55	55
Gamma	1.5	1.5
Hue	8	8
Saturation	84	84
NGENUITY Factor	N/A	3

N1.4 = NGENUITY version 1.4; N1.5 = NGENUITY version 1.5; N/A = not available in this version. All parameters were identical between versions except the NGENUITY Factor, available only in version 1.5.

**Table 2:** Baseline Patient and Surgical Characteristics by Group.

Characteristic	Microscope	N1.4	N1.5	*p* Value
Patients, n (eyes)	16 (21)	24 (25)	20 (21)	
Age (years, mean ± SD)	70.8 ± 10.1	72.4 ± 9.7	70.7 ± 9.8	0.809
Sex (female/male)	8/8	13/11	10/10	0.951
Emery–Little grade 1/2/3 (eyes)	3/9/9	3/15/7	5/13/3	—
Preoperative BCVA (logMAR, mean ± SD)	0.30 ± 0.25	0.24 ± 0.27	0.20 ± 0.23	0.516

N1.4 = NGENUITY version 1.4; N1.5 = NGENUITY version 1.5; BCVA = best-corrected visual acuity; SD = standard deviation. Sex is reported per patient, whereas cataract grade is reported per eye.

## Data Availability

The anonymized eye-level dataset supporting the findings of this study (Supplementary S1 and S1b) and the complete R analysis script reproducing all statistical models reported in this manuscript (Supplementary S2) are provided as Supplementary Materials with this submission. The underlying surgical video recordings are not publicly available due to patient privacy but are available from the corresponding author on reasonable request.

## Supplementary Materials

The following supporting information can be downloaded at: https://www.mdpi.com/article/10.3390/jcm15197430/s1, Supplementary S1. Anonymized dataset; Supplementary S1b. CCR triplicate raw; Supplementary S2. R analysis script; Figure S1. Patient screening, exclusion, and inclusion flow diagram across the three visualization modalities.
